# Persistent Post-Concussion Syndrome Following Motor Vehicle Collision: A 27-Month Longitudinal Case Study of Multimodal Rehabilitation

**DOI:** 10.3390/neurosci7050098

**Published:** 2026-08-31

**Authors:** Kenneth Jay, Mary Shaya, Jonathan Walker

**Affiliations:** 1 Department of Research, Ethos Health Group, Ocala, FL 34471, USA; drshaya@ethoshealthgroup.com (M.S.); drwalker@ethoshealthgroup.com (J.W.); 2 Department of Functional Neurology, Carrick Institute, Cape Canaveral, FL 32920, USA; 3 Department of Research, Cervello A/S, 4000 Roskilde, Denmark; 4 Department of Medicine, University of Central Florida, Orlando, FL 32816, USA

**Keywords:** post-concussion syndrome, traumatic brain injury, multimodal rehabilitation, neurorehabilitation, longitudinal outcomes, CARE guidelines

## Abstract

Persistent post-concussion syndrome (PPCS) develops in 10 to 30 percent of patients following mild traumatic brain injury (mTBI), yet the most effective management approach remains an open question. This report describes a 37-year-old female who sustained a motor vehicle collision and was followed prospectively over 27 months. Structured evaluation at baseline identified dysfunction across oculomotor, vestibular, autonomic, cognitive, and neurophysiological domains. An intensive multimodal rehabilitation programme was associated with an 86 percent reduction in symptom burden (Post-Concussion Symptom Scale: 80 to 11) by the six-month mark. Thereafter, symptoms gradually returned in spite of continued treatment, and at 27 months the patient still reported moderate-to-severe headache (6 to 7 out of 10), persistent dizziness (5 to 6 out of 10), and meaningful functional limitations. The trajectory observed here, marked by initial gains followed by progressive relapse, illustrates the fluctuating and often incomplete recovery seen in a subgroup of PPCS patients. Clinicians should anticipate this pattern and frame outcome expectations accordingly, while building sustained, adaptive management strategies into long-term care plans.

## 1. Introduction

Motor vehicle collisions are among the most frequent causes of mild traumatic brain injury (mTBI), a condition estimated to affect roughly 42 million people worldwide every year [1]. For the majority, recovery is straightforward and complete within a matter of weeks. A substantial minority, however, somewhere between 10 and 30 percent of those injured, go on to develop persistent post-concussion syndrome (PPCS), defined by cognitive, physical, and emotional symptoms that continue beyond three months after the injury [2,3]. Managing PPCS is genuinely difficult: presentations vary widely, the underlying pathophysiology is not fully understood, and the evidence base for treatment remains limited [4].

We use the term persistent post-concussion syndrome (PPCS) throughout this report to denote symptoms persisting beyond three months following mTBI. We acknowledge that this terminology remains subject to ongoing debate; some authors prefer post-concussive symptoms, mild traumatic brain injury with persistent symptoms, or mild traumatic brain injury-related disorder, to avoid implying a discrete and homogeneous syndrome [5].

### 1.1. Pathophysiology of Persistent Post-Concussion Syndrome

What drives PPCS at a biological level is still being explored, though it is clear that multiple processes interact. In the immediate aftermath of mTBI, a neurometabolic cascade unfolds, involving ionic flux disruption, glutamate release, mitochondrial dysfunction, oxidative stress, and altered cerebral blood flow, all occurring in rapid succession [6]. In most cases, these disturbances settle within days to weeks. When they do not, persistent neuroinflammation, ongoing mitochondrial impairment, autonomic dysregulation, and disrupted neural network connectivity are thought to keep symptoms alive [7,8].

Neuroimaging has shed further light on the picture. Studies consistently identify disruptions in large-scale brain networks after mTBI, with the default mode network (DMN), salience network, and executive control network all showing abnormalities [9]. The DMN, anchored in the medial prefrontal cortex, posterior cingulate cortex, and angular gyrus, underpins self-referential thought and is frequently compromised in PPCS [10]. Dysfunction within the salience network, which involves the anterior insula and dorsal anterior cingulate cortex, appears to drive attentional difficulties and may amplify symptom perception [11]. Meanwhile, changes in the executive control network, centered on the dorsolateral prefrontal cortex, track closely with the cognitive complaints that patients most commonly report [12]. The NERD model, which conceptualizes persistent post-concussion symptoms as a product of reflex circuit dysfunction operating at a systems level, offers a complementary framework for integrating these multidomain findings into a coherent clinical picture [13].

### 1.2. Multimodal Rehabilitation Approaches

Because PPCS is multifaceted, there is growing consensus that no single intervention is sufficient. Contemporary management therefore draws on a range of modalities tailored to each patient’s symptom profile [14,15]. Vestibular and oculomotor rehabilitation directly target balance disturbance and visual tracking difficulties, both of which are common following mTBI [16,17]. Graduated aerobic exercise has accumulated a meaningful evidence base, with controlled studies demonstrating improvements in symptom burden and neurocognitive performance [18]. Cognitive rehabilitation, delivered through structured compensatory strategies and direct training, helps patients manage attention, memory, and executive function deficits [19].

Emerging adjunctive therapies are also attracting interest. Transcranial photobiomodulation (tPBM) uses near-infrared light to stimulate mitochondrial function and has shown encouraging results in small studies of chronic mTBI [20]. Transcutaneous vagus nerve stimulation (tVNS) modulates autonomic tone and neuroinflammatory pathways and has been explored as a tool for reducing hyperarousal and autonomic dysfunction in patients with overlapping mTBI and post-traumatic stress [21]. Autonomic biofeedback, particularly heart rate variability (HRV) training, targets the autonomic instability that is well documented in concussed populations [22].

Despite this expanding toolkit, longitudinal data on how PPCS patients actually fare over extended follow-up periods remain sparse. The possibility of delayed relapse following early improvement has not been systematically characterised, and few published case reports document more than twelve months of structured outcome monitoring. The case described here spans 27 months of structured observation and serial objective measurement, offering a detailed view of both the initial response to intensive multimodal rehabilitation and the subsequent relapse.

The objectives of this case report are threefold: (1) to document the 27-month clinical course of a patient with PPCS who underwent comprehensive multimodal rehabilitation; (2) to describe the assessment and treatment programme in sufficient detail to support clinical replication; and (3) to discuss the implications of the biphasic recovery trajectory, including considerations for monitoring, patient counselling, and long-term management planning.

## 2. Materials and Methods

This report is a retrospective observational case study prepared in accordance with the CARE (CAse REport) guidelines for transparent case reporting [23]. The case was identified from clinical records at Ethos Health Group, Ocala, Florida. All procedures were conducted in accordance with the ethical principles of the Declaration of Helsinki. The mandatory ethics statement is provided in the Informed Consent Statement section below.

### 2.1. Case Presentation

#### 2.1.1. Patient Information and Clinical History

The patient is a 37-year-old right-handed female with no prior history of concussion, neurological illness, or psychiatric diagnosis. In March 2022, she was the restrained driver in a motor vehicle collision in which her vehicle was struck on the driver’s side at approximately 35 mph. She did not lose consciousness but was immediately confused and disoriented, with difficulty processing her surroundings. Concurrent injuries included rib fractures and a scapular fracture. In the 17 months that followed, from March 2022 through August 2023, she received various treatments in the community, none of which resolved her symptoms. She was ultimately referred to the neurorehabilitation clinic in August 2023, at which point her presentation met diagnostic criteria for PPCS [2,3].

At the time of referral, she described daily headache rated 8 out of 10, persistent dizziness rated 7 out of 10, cognitive slowing, visual disturbances, cervical pain, fatigue, and emotional lability. She reported being unable to work, requiring assistance with household management and childcare, and having withdrawn substantially from social activities. In her own words: “I feel like I am living in a fog that never lifts. I cannot think clearly, I cannot function normally, and I have lost who I was before the accident.”

#### 2.1.2. Initial Clinical Presentation

Neurological examination at the August 2023 assessment identified oculomotor dysfunction with impaired smooth pursuit, saccadic intrusions, and convergence insufficiency. Vestibular testing revealed abnormal head impulse responses bilaterally and positive dynamic visual acuity testing. Cervical examination demonstrated restricted range of motion with myofascial tenderness throughout the suboccipital and upper trapezius musculature. Neuropsychological screening was consistent with deficits in processing speed, working memory, and sustained attention. Autonomic assessment using heart rate variability (HRV) analysis indicated significant parasympathetic withdrawal and sympathetic dominance at rest. Olfactory function was reduced bilaterally on standardized testing.

Formal audiological assessment was not conducted at this time. Hearing loss, which can accompany vestibular dysfunction following acceleration-deceleration injury, cannot therefore be excluded as a contributing factor to the patient’s symptom profile. This represents a gap in the initial diagnostic evaluation.

### 2.2. Comprehensive Diagnostic Assessment

#### 2.2.1. Neuroimaging Findings

MRI of the brain with FLAIR sequences identified a T2/FLAIR signal change in the left frontal white matter, consistent with post-traumatic axonal injury. No hemorrhage, contusion, or space-occupying lesion was identified. MRI of the cervical spine demonstrated a C5–6 disc herniation with mild foraminal encroachment, correlating with the patient’s cervicogenic symptom component. These structural findings were interpreted in the context of her clinical presentation and were consistent with published neuroimaging data in chronic mTBI populations [24,25].

#### 2.2.2. Oculomotor Assessment

Detailed oculomotor evaluation using infrared videonystagmography documented impaired smooth pursuit (gain 0.72 bilaterally; normal 0.90 or above), hypometric horizontal saccades (accuracy 84 percent; normal 95 percent or above), and a convergence near point of 12 cm (normal 6 cm or less). The Vestibular/Ocular Motor Screening (VOMS) battery yielded a provocation score of 7 out of 10 on oculomotor tasks, well above the symptom threshold. These findings pointed to central oculomotor pathway disruption rather than peripheral ocular pathology, consistent with prior reports of oculomotor dysfunction in PPCS [16,17].

#### 2.2.3. Vestibular Function Testing

Comprehensive vestibular assessment included video head impulse testing (vHIT), computerized dynamic posturography, and the Dizziness Handicap Inventory (DHI). vHIT revealed corrective saccades on bilateral horizontal head impulses, indicating semicircular canal hypofunction. Dynamic posturography demonstrated abnormal performance on conditions requiring vestibular input (Sensory Organisation Test conditions 5 and 6). The DHI total score was 64 out of 100, placing the patient in the severe handicap range. VOMS vestibular provocation score was 8 out of 10. Taken together, these results indicate both peripheral and central vestibular involvement, a pattern that has been associated with prolonged recovery in mTBI [16].

#### 2.2.4. Neurophysiological Assessment

Quantitative EEG (qEEG) mapping demonstrated diffuse slowing with excess delta and theta activity in frontal and temporal regions, alongside reduced alpha coherence between prefrontal and parietal sites. These patterns are consistent with post-traumatic dysregulation of corticothalamic networks and have been reported in chronic mTBI with persistent cognitive symptoms [25]. Event-related potential (ERP) testing showed prolonged P300 latency (412 ms; normal 380 ms or less) and reduced P300 amplitude, indicating slowed cortical information processing, an objective correlate of the patient’s subjective cognitive complaints.

#### 2.2.5. Autonomic Function Assessment

Five-minute resting HRV analysis in the supine position yielded markedly reduced time- and frequency-domain indices: SDNN 18 ms (normal 50 ms or above), RMSSD 14 ms (normal 30 ms or above), LF/HF ratio 4.8 (normal 1.5 to 2.0), and total power 312 ms squared (normal 1000 ms squared or above). These values indicated pronounced sympathetic dominance and impaired vagal modulation, consistent with the autonomic dysregulation documented in concussed populations [22]. Orthostatic testing revealed a 22 mmHg drop in systolic blood pressure on standing, meeting criteria for orthostatic hypotension and suggesting additional cardiovascular autonomic compromise.

#### 2.2.6. Cognitive Performance Testing

ImPACT neurocognitive testing at baseline produced the following composite scores: Verbal Memory 72 percent (normative mean 85 percent), Visual Memory 68 percent (normative mean 80 percent), Processing Speed 38 correct symbols (normative mean 50 or above), and Reaction Time 382 ms (normative threshold 350 ms or less). The Standardised Assessment of Concussion (SAC) yielded a total score of 22 out of 30, below the established threshold of 27 for normal performance. Taken together, these results confirm clinically significant impairment across multiple cognitive domains, particularly processing speed and reaction time, domains known to be especially sensitive to mTBI [6,26].

#### 2.2.7. Olfactory Function Testing

The University of Pennsylvania Smell Identification Test (UPSIT) yielded a score of 28 out of 40, placing the patient at the low-average range (18th percentile) for her age and sex, suggesting subclinical olfactory reduction. Post-traumatic olfactory dysfunction following acceleration-deceleration injury has been associated with orbitofrontal cortex and olfactory bulb involvement [13].

### 2.3. Therapeutic Intervention

A comprehensive multimodal rehabilitation program was initiated in September 2023, delivered three times weekly across the following domains:

- TBI Rehabilitation: Structured cognitive and physical rehabilitation targeting post-concussive symptom clusters, with progressive loading based on symptom response.
- Oculomotor Therapy: Graded pursuit and saccadic training, convergence exercises, and visual–vestibular integration tasks to address documented oculomotor deficits.
- Vestibular Therapy: Canalith repositioning, gaze stabilization exercises, and dynamic balance training targeting both peripheral and central vestibular dysfunction.
- Autonomic Biofeedback: HRV-guided biofeedback training to restore parasympathetic tone and reduce sympathetic dominance, three sessions weekly.
- Cognitive Rehabilitation: Compensatory strategy training and direct cognitive exercises addressing processing speed, working memory, and sustained attention.
- Transcranial Photobiomodulation (tPBM): Near-infrared light delivered transcranially at 810 nm, targeting frontal and temporal regions to support mitochondrial function and reduce neuroinflammation [20].
- Alpha Wave Neurostimulation: Non-invasive neurostimulation targeting alpha frequency dysregulation identified on qEEG, with the goal of restoring cortical oscillatory patterns.
- Transcutaneous Vagus Nerve Stimulation (tVNS): Non-invasive auricular vagal stimulation to modulate autonomic tone and attenuate neuroinflammatory signaling [21].
- Pain Management: Pharmacological support included gabapentin, topiramate, and cyclobenzaprine. Thoracic medial branch blocks were administered for refractory axial pain.

The programme was monitored continuously, with objective reassessment at each reassessment visit and adjustments made in response to symptom changes. The patient attended all scheduled sessions throughout the observation period.

All modalities were initiated concurrently at programme outset and delivered three times weekly throughout the 27-month observation period, with the intensity and focus of each modality adjusted at each reassessment visit based on symptom response and objective findings. Session parameters were as follows: transcranial photobiomodulation (tPBM) using an 810 nm near-infrared device, 20 min sessions targeting frontal and temporal regions; transcutaneous vagus nerve stimulation (tVNS) delivered via the InTENSity Micro Combo device at 10 Hz, 20 microsecond pulse width, 20 min sessions at subthreshold current intensity; cranial electrotherapy stimulation (Alpha Stim AID) at 0.5–5 Hz, 0–500 microamps, 20 min sessions; heart rate variability (HRV) biofeedback via the Thought Technology BioGraph Infinity platform; oculomotor rehabilitation guided by the RightEye eye-tracking system; and structured cognitive rehabilitation via HappyNeuron Pro. Exact dosing parameters for vestibular rehabilitation and oculomotor exercises were not individually documented per session.

**Figure 1:**
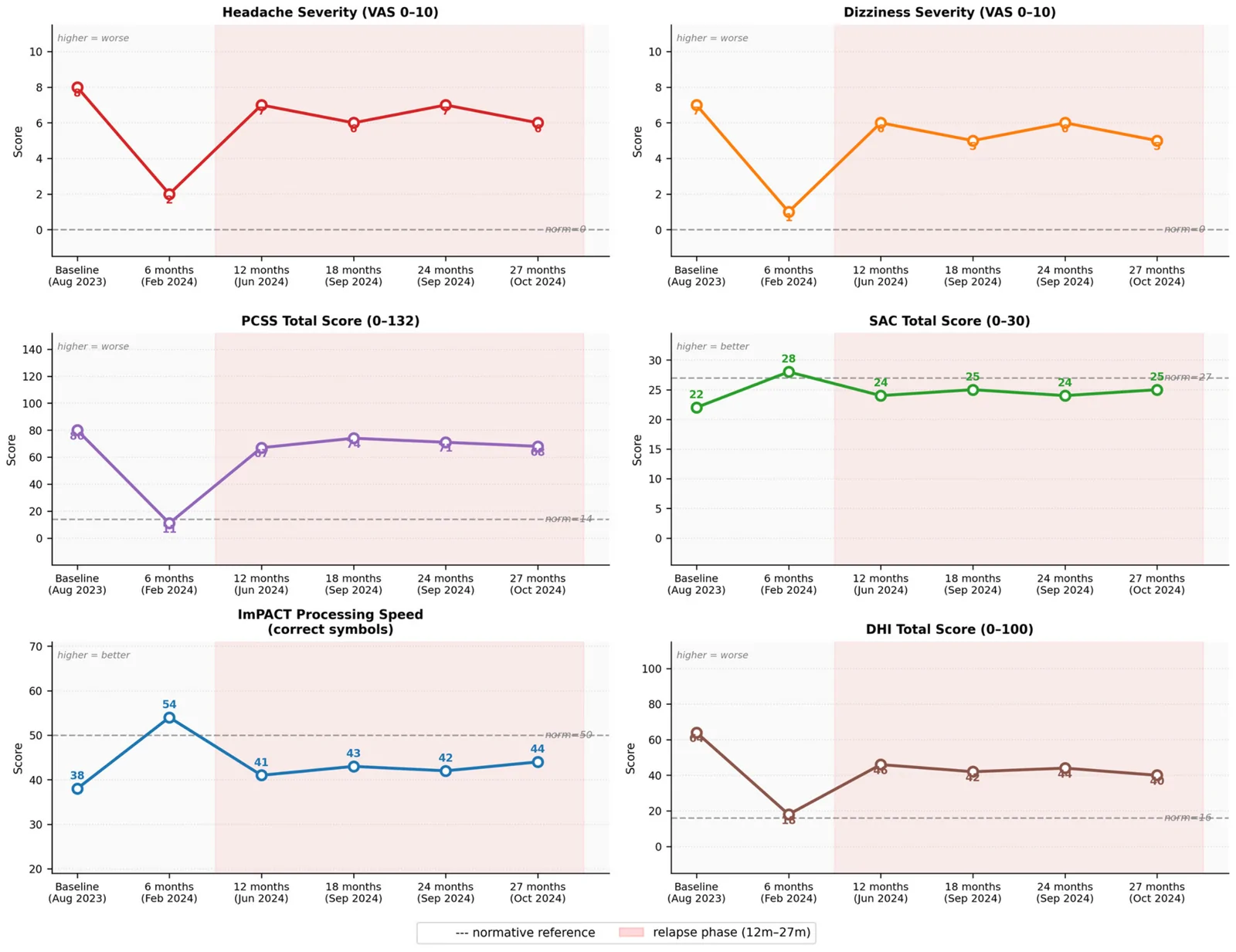
Symptom trajectory across six assessment timepoints over 27 months. The figure displays standardised scores for six clinical domains: headache severity (Visual Analog Scale, 0 to 10), dizziness severity (Visual Analog Scale, 0 to 10), Post-Concussion Symptom Scale (PCSS) total score (0 to 132), Standardised Assessment of Concussion (SAC) total score (0 to 30), ImPACT Processing Speed (correct symbols), and Dizziness Handicap Inventory (DHI) total score (0 to 100). Timepoints are: Baseline (August 2023), 6 months (February 2024), 12 months (June 2024), 18 months (September 2024), 24 months (September 2024), and 27 months (October 2024). The trajectory illustrates substantial symptom reduction at six months followed by progressive relapse across most domains from twelve months onward.

No adverse events or unexpected side effects were reported by the patient during the observation period. Tolerability was monitored at each reassessment visit through structured symptom enquiry, and no treatment was modified or discontinued for safety reasons (Figure 1).

## 3. Results

Table 1 shows the timeline of key clinical events.

### 3.1. Six-Month Assessment (February 2024; 23 Months Post-Injury; 6 Months Post-Rehabilitation Initiation)

At six months, the response to treatment was striking. The PCSS total score fell from 80 to 11, an 86 percent reduction, and headache intensity dropped from 8 out of 10 to 2 out of 10. Dizziness improved from 7 out of 10 to 1 out of 10. SAC performance recovered to 28 out of 30, within the normal range. ImPACT Processing Speed reached 54 correct symbols and Reaction Time improved to 318 ms, both within normative limits. The DHI score fell to 18 out of 100, at the boundary of the mild handicap range. The patient reported being able to resume light domestic activities and part-time childcare and described her quality of life as significantly improved. HRV indices showed meaningful recovery: SDNN increased to 38 ms and RMSSD to 26 ms, reflecting partial restoration of vagal tone.

### 3.2. Twelve-Month Assessment (June 2024; 27 Months Post-Injury; 12 Months Post-Rehabilitation Initiation)

By twelve months, however, the clinical picture had deteriorated markedly. The PCSS total score rose to 67 out of 132, headache returned to 7 out of 10, and dizziness climbed back to 6 out of 10. SAC performance declined to 24 out of 30. ImPACT Processing Speed regressed to 41 correct symbols and Reaction Time extended to 361 ms, both outside normative limits once again. The DHI score rose to 46 out of 100. The patient reported requiring significant assistance with household management and childcare and had been unable to return to any form of employment. She described the relapse as devastating and expressed frustration that gains achieved earlier in the year had not been sustained.

No specific precipitating factors, such as concurrent illness, new medication, significant psychosocial stressor, or change in life circumstances, were documented in the clinical record at the time of this deterioration. The absence of contextual information about potential triggers limits the conclusions that can be drawn regarding the mechanism of the relapse.

### 3.3. Long-Term Follow-Up (18–27 Months Post-Rehabilitation Initiation; September–October 2024; 31–32 Months Post-Injury)

Assessments at 18, 24, and 27 months post-initiation of rehabilitation (September and October 2024) confirmed that the patient had stabilized at a level substantially below her six-month peak. Headache ranged between 6 and 7 out of 10 across these visits; dizziness ranged between 5 and 6 out of 10. SAC scores fluctuated between 24 and 25 out of 30. ImPACT Processing Speed remained in the 41 to 44 correct symbol range and Reaction Time between 350 and 361 ms. The DHI total score ranged from 40 to 46 out of 100. GAD-7 and PHQ-9 scores indicated moderate anxiety (10 to 11 out of 21) and mild-to-moderate depression (8 to 10 out of 27), both elevated relative to the six-month nadir. Functional status was rated 5 to 6 out of 10, compared with 8 out of 10 at six months and 3 out of 10 at baseline. The patient remained unable to work and continued to require daily assistance (Table 2).

No repeat neuroimaging or quantitative electroencephalography was performed during the follow-up period. The absence of serial neurophysiological data limits the ability to correlate the clinical relapse with any structural or functional brain changes and represents a gap in the longitudinal documentation.

### 3.4. Patient Perspective

Across the follow-up period, the patient provided candid accounts of her lived experience that add important qualitative context to the objective data. At baseline, she described feeling trapped in a body that no longer worked the way it should, with a pervasive sense of cognitive fog and physical exhaustion that made even simple tasks feel overwhelming. The six-month improvement brought genuine relief: “For the first time since the accident, I could see a future. I felt like myself again, not completely, but enough to hope.”

The relapse at twelve months was, in her words, the hardest thing she had been through, worse in some ways than the accident itself, because she had already started to get better. She described a return of cognitive fog, increased light and noise sensitivity, emotional dysregulation, and a profound loss of confidence in her own recovery. At 27 months, she remained cautiously engaged with ongoing treatment but acknowledged that her expectations had shifted: “I have had to accept that this may be my new normal, at least for now. I still hope things will improve, but I have stopped waiting for the day I wake up and feel like I did before.”

## 4. Discussion

The central finding of this case is a biphasic recovery trajectory: a period of dramatic improvement in the first six months of multimodal rehabilitation, followed by substantial relapse that persisted through to the 27-month endpoint. At six months, an 86 percent reduction in PCSS score was observed, normalisation of SAC performance, and recovery of ImPACT indices into normative ranges suggested that the patient had achieved near-complete symptomatic resolution. Yet by twelve months, most of these gains had been reversed. This pattern, encouraging early response followed by late deterioration, is not widely represented in the PPCS case literature, which tends to report either sustained improvement or persistent plateau rather than the oscillating course seen here.

The clinical significance of this trajectory extends beyond the individual case. It challenges the assumption that early objective improvement in PPCS reliably predicts long-term outcome. It also raises questions about the durability of multimodal rehabilitation gains and the mechanisms by which relapse occurs. For clinicians, these findings raise the possibility that patients who respond well to initial treatment should not be discharged from active follow-up, and management plans should include explicit provisions for monitoring and responding to late deterioration.

The 10 to 30 percent prevalence of PPCS following mTBI is well established [2,3], and the heterogeneous, multidomain nature of the condition has been described in detail [4,14]. What is less well characterized is the long-term trajectory in patients who receive structured multimodal rehabilitation. Most published case reports and small series follow patients for three to twelve months; 27-month longitudinal data of this granularity are uncommon in the literature.

The initial treatment response observed here is consistent with evidence supporting vestibular rehabilitation [16,27], oculomotor therapy [17], and graduated aerobic exercise [18,28] in PPCS. Leddy et al. (2018) demonstrated that exercise-based interventions can accelerate symptom resolution, and the early gains in this case align with that finding [28]. Similarly, the improvements in HRV indices following autonomic biofeedback are consistent with published data on HRV training in concussed populations [22].

The relapse phase is harder to map onto existing literature, largely because few studies have tracked PPCS patients beyond twelve months with serial objective measures. Theadom et al. (2016) reported that a significant proportion of mTBI patients continue to experience problems at one year [29], and Venkatesan et al. (2015) documented persistent alterations in resting-state functional connectivity in chronic TBI [25], findings that may provide a neurobiological substrate for the relapse observed here. The cogniphobia framework described by Silverberg et al. (2017) offers another lens: fear-avoidance behaviours and catastrophising cognitions may have contributed to symptom amplification following the twelve-month downturn [30].

In this patient, GAD-7 and PHQ-9 scores at twelve months indicated moderate anxiety (GAD-7: 10 out of 21) and mild-to-moderate depression (PHQ-9: 9 out of 27). These findings are consistent with, though not diagnostic of, a fear-avoidance response to symptom relapse, and suggest that psychological factors may have contributed to the maintenance of the clinical downturn at this stage.

The structural findings on MRI, specifically the left frontal T2/FLAIR signal change and C5–6 disc herniation, are relevant to both the initial severity and the incomplete recovery. White matter signal changes of this type have been associated with worse long-term outcomes in mTBI [24], and cervicogenic contributions to headache and dizziness are well recognized [31,32]. The persistence of cervical pathology despite treatment may have acted as a sustained nociceptive driver, limiting the ceiling of achievable recovery regardless of how well the central post-concussive processes were addressed.

### Mechanistic Considerations

Several mechanisms may account for the relapse trajectory. First, the neuroinflammatory model of PPCS posits that initial treatment can suppress but not fully extinguish inflammatory cascades; re-exposure to physical or psychological stressors may then rekindle these processes [7,8]. Second, the persistent autonomic dysregulation documented throughout this case, with only partial HRV recovery even at the six-month peak, suggests that the autonomic nervous system remained vulnerable to decompensation [22]. Third, the qEEG abnormalities, specifically excess delta and theta with reduced alpha coherence, did not fully normalize during the observation period, indicating that corticothalamic network dysregulation persisted at a subclinical level even when symptoms were at their lowest [25]. The NERD model framework is instructive here: reflex circuit dysfunction operating across multiple systems may explain why symptomatic improvement did not correspond to full neurophysiological normalization [33].

The tPBM and tVNS components of the treatment protocol deserve specific comment. Both modalities target mechanisms, namely mitochondrial dysfunction and autonomic dysregulation, that are implicated in PPCS persistence [20,21]. The partial and temporary nature of the response in this case suggests that either the dosing parameters were suboptimal, the treatment window was insufficient, or these mechanisms represent only part of a more complex pathophysiological picture. Controlled trials with standardized protocols are needed before firm conclusions can be drawn about the contribution of these modalities to long-term outcomes.

The cervical spine findings add another layer of mechanistic complexity. Weightman et al. (2010) outlined physical therapy recommendations specifically for service members with mTBI and cervical involvement [32], and Ellis et al. (2015) proposed a classification system that distinguishes cervicogenic from physiological and vestibulo-ocular post-concussion disorders [31]. In this patient, the C5–6 disc herniation with foraminal encroachment likely contributed to a cervicogenic headache component that was partially responsive to medial branch blocks but not fully resolved. This unresolved cervical pathology may have functioned as a persistent pain generator that undermined the gains achieved through other rehabilitation modalities.

Beyond neurobiological mechanisms, the role of psychosocial stressors in perpetuating the relapse cannot be excluded. The patient carried substantial caregiving responsibilities for dependent family members throughout the observation period, a burden that may have contributed to physiological allostatic load and limited her capacity for recovery. Formal assessment of life stressors at the time of relapse was not conducted, representing a gap in the clinical data. No treatment changes were made specifically in response to the relapse.

This case carries several practical messages for clinicians managing PPCS. The first is the importance of comprehensive baseline assessment. The breadth of dysfunction documented here, spanning oculomotor, vestibular, autonomic, cognitive, neurophysiological, and olfactory domains, would not have been captured by a standard clinical evaluation. In this patient, each identified domain appeared to warrant a targeted intervention; failure to assess any one of them may have left a potential driver of symptoms unaddressed. Whether targeted intervention across all domains is uniformly necessary—or whether some patients recover without it—remains an open empirical question.

The second implication concerns the design of follow-up protocols. The relapse in this case occurred between the six- and twelve-month assessments, a window during which the patient might have been considered stable or improving based on earlier data. More frequent monitoring, particularly in the six-to-twelve-month period, could allow earlier detection of deterioration and more timely therapeutic adjustment. The use of brief, validated tools such as the PCSS and SAC at regular intervals would add little burden while providing meaningful early-warning data.

Third, this case underlines the value of realistic patient counselling. The patient’s accounts reveal how the relapse, experienced after a period of genuine improvement, was psychologically more devastating than the initial injury. Preparing patients for the possibility of a non-linear course and framing early gains as encouraging but not necessarily permanent may reduce the psychological impact of setbacks and help maintain engagement with treatment. The cogniphobia literature suggests that how patients interpret and respond to symptom fluctuation can itself influence outcome [30].

Finally, the case raises the question of what constitutes adequate treatment duration for PPCS. Current guidelines generally recommend active rehabilitation for three to six months [14,15,34,35]. The data presented here suggest that, for at least some patients, this timeframe is insufficient and that ongoing, adaptive management over a much longer horizon may be necessary.

The primary strength of this report is the length and granularity of the follow-up. Twenty-seven months of serial objective assessment across multiple validated instruments provides a level of longitudinal detail that is rare in the PPCS case literature. The use of standardized tools (ImPACT, SAC, PCSS, VOMS, DHI, GAD-7, PHQ-9, HRV analysis, qEEG) across all timepoints allows direct comparison and trend analysis. The inclusion of the patient’s own narrative adds qualitative depth that purely quantitative reports cannot provide.

The limitations are inherent to the case report design. Findings from a single patient cannot be generalized, and the absence of a control condition means that the observed trajectory cannot be attributed definitively to the interventions delivered. The multimodal nature of the treatment protocol makes it impossible to isolate the contribution of any individual component. Reporting bias cannot be excluded. These constraints are acknowledged; they are also characteristic of all single-case reports and do not diminish the descriptive and hypothesis-generating value of the data presented.

Additional limitations warrant explicit acknowledgement. The MRI finding of left frontal white matter T2/FLAIR signal change, together with the persistent qEEG abnormalities, raises the possibility that this patient’s injury was more severe than a typical uncomplicated concussion. This qualification limits the generalisability of these findings to the broader PPCS population. No repeat neuroimaging was performed during the follow-up period, meaning that any progression or resolution of the white matter change cannot be assessed. No serial quantitative EEG was conducted after the baseline assessment, limiting interpretation of the neurophysiological recovery trajectory. Hearing was not formally assessed, as noted in the Case Presentation.

The extent to which the multimodal programme contributed to outcomes, whether at the six-month peak or during the relapse phase, cannot be determined from a single case. Randomised controlled trials comparing individual treatment components and multimodal combinations are needed to establish whether this programme, or any specific component, has efficacy beyond natural history. Given the resource intensity of the protocol described here, such trials should also evaluate feasibility and cost-effectiveness across different healthcare settings.

## 5. Conclusions

This 27-month longitudinal case study documents a biphasic recovery trajectory in a patient with PPCS following motor vehicle collision: substantial early improvement under intensive multimodal rehabilitation, followed by progressive relapse that persisted through the end of the observation period. At 27 months, the patient remained significantly symptomatic and functionally limited despite continued treatment.

The case highlights several points of clinical relevance. Comprehensive multimodal assessment is essential for identifying the full range of post-concussive dysfunction. Early treatment response, while encouraging, does not guarantee durable recovery. Long-term follow-up with structured monitoring is necessary to detect and respond to late deterioration. And realistic, ongoing patient counselling, acknowledging the possibility of a fluctuating, prolonged course, is an integral component of responsible PPCS management.

Prospective longitudinal studies with larger samples and standardized protocols are needed to determine how frequently this biphasic pattern occurs, which patient characteristics predict it, and whether modified treatment approaches can improve long-term durability of gains.

Finally, this case highlights the importance of considering treatment burden and patient life context in PPCS management. An intensive three-times-weekly schedule maintained over 27 months is substantial, and the possibility that treatment fatigue, or the combined demands of rehabilitation and caregiving responsibilities, contributed to the relapse cannot be excluded. Clinicians should monitor patient-reported treatment burden alongside clinical outcomes and be prepared to adapt schedules as needed to maintain patient engagement and minimise iatrogenic stress.

It should be noted that maintaining a strictly uninterrupted three-times-weekly schedule across 27 months is clinically unlikely and was not expected in practice. The rehabilitation schedule described in this report was adapted as needed in response to life events, including illness, holiday periods, family obligations, and other personal circumstances; the programme frequency cited represents the intended clinical protocol rather than a continuously enforced, invariant timetable.

## Figures and Tables

**Table 1:** Timeline of key clinical events. The table presents a chronological summary of the major clinical milestones from the index motor vehicle collision in March 2022 through the 27-month follow-up endpoint in October 2024, including the pre-referral period, initial presentation, rehabilitation initiation, and serial follow-up assessments. Each row includes the date, event description, key findings or interventions, and clinical significance.

Date	Event	Key Findings/Interventions	Clinical Significance
March 2022	Motor Vehicle Collision	Restrained driver; struck driver-side at approximately 35 mph; no loss of consciousness; immediate confusion, disorientation, difficulty processing information; concurrent rib fractures and scapular fracture.	Index mTBI event.
March 2022 to August 2023	Pre-Referral Period (17 months)	Persistent symptoms despite community treatment; ongoing headache, dizziness, cognitive difficulties, visual disturbances, neck pain, fatigue, and emotional lability. Note (R1): Specific community treatments were not documented in the referral record.	Failure to resolve with prior care; established chronicity.
August 2023	Initial Presentation to Neurorehabilitation Clinic	Headache 8/10; dizziness 7/10; SAC 22/30; PCSS 130/162; Processing Speed 38; Reaction Time 382 ms; MRI: left frontal T2/FLAIR signal change, C5–6 disc herniation; oculomotor, vestibular, autonomic, and olfactory deficits documented.	Comprehensive baseline established; PPCS confirmed across multiple domains.
September 2023	Multimodal Rehabilitation Initiated	TBI rehabilitation, oculomotor therapy, vestibular therapy, autonomic biofeedback, cognitive rehabilitation, transcranial photobiomodulation, alpha wave neurostimulation, transcutaneous vagus nerve stimulation, pain management (gabapentin, topiramate, cyclobenzaprine, thoracic medial branch blocks).	Intensive multimodal programme commenced three times weekly.
February 2024	Six-Month Assessment	PCSS decreased 86 percent (80 to 11); headache 2/10; dizziness 1/10; SAC 28/30; improved functional capacity; HRV indices partially restored.	Substantial multidomain improvement; most favourable outcome point.
June 2024	Twelve-Month Assessment	PCSS increased to 67/132; headache returned to 7/10; dizziness 6/10; SAC declined to 24/30; processing speed and reaction time regressed; required significant assistance with household management and childcare.	Significant symptom recurrence despite continued treatment.
September to October 2024	Long-Term Follow-Up (18, 24, and 27 Months Post-Initial Evaluation)	Headache 6 to 7/10; dizziness 5 to 6/10; ongoing cognitive complaints and visual disturbances; persistent functional limitations; unable to return to pre-injury independence.	Incomplete recovery confirmed at 27-month endpoint; fluctuating course established.

Note: All dates and timepoints correspond directly to clinical documentation. Patient identifying information has been anonymised in accordance with CARE guidelines and the HIPAA Safe Harbor method.

**Table 2:** Standardised assessment scores across follow-up timepoints. The table presents quantitative scores from validated assessment instruments administered at each of six timepoints: baseline (August 2023), 6 months (February 2024), 12 months (June 2024), 18 months (September 2024), 24 months (September 2024), and 27 months (October 2024). Instruments include the Post-Concussion Symptom Scale (PCSS), Standardised Assessment of Concussion (SAC), ImPACT neurocognitive battery, Vestibular/Ocular Motor Screening (VOMS), Dizziness Handicap Inventory (DHI), Generalized Anxiety Disorder 7-item scale (GAD-7), Patient Health Questionnaire-9 (PHQ-9), and functional status rating. Normative reference values are provided where applicable.

Assessment Tool	Baseline (August 2023)	6 Months (February 2024)	12 Months (June 2024)	18 Months (September 2024)	24 Months (September 2024)	27 Months (October 2024)	Normative Reference
PCSS Total (0 to 132)	80	11	67	74	71	68	Below 14 (asymptomatic)
Headache (VAS 0 to 10)	8/10	2/10	7/10	6/10	7/10	6/10	0
Dizziness (VAS 0 to 10)	7/10	1/10	6/10	5/10	6/10	5/10	0
SAC Total (0 to 30)	22	28	24	25	24	25	27 or above
ImPACT Verbal Memory (%)	72	88	78	79	77	80	85 or above
ImPACT Visual Memory (%)	68	84	71	73	70	74	80 or above
ImPACT Processing Speed (correct)	38	54	41	43	42	44	50 or above
ImPACT Reaction Time (ms)	382	318	361	354	358	350	350 or less
VOMS Oculomotor (0 to 10 VAS)	7	2	5	4	5	4	0 to 2
VOMS Vestibular (0 to 10 VAS)	8	2	6	5	6	5	0 to 2
DHI Total (0 to 100)	64	18	46	42	44	40	Below 18 (mild)
GAD-7 (0 to 21)	16	6	12	11	12	10	Below 5 (minimal)
PHQ-9 (0 to 27)	14	5	10	9	10	8	Below 5 (minimal)
Functional Status (0 to 10)	3	8	5	5	5	6	9 to 10

Abbreviations: PCSS = Post-Concussion Symptom Scale; SAC = Standardised Assessment of Concussion; ImPACT = Immediate Post-Concussion Assessment and Cognitive Testing; VOMS = Vestibular/Ocular Motor Screening; DHI = Dizziness Handicap Inventory; GAD-7 = Generalized Anxiety Disorder 7-item scale; PHQ-9 = Patient Health Questionnaire-9; VAS = Visual Analog Scale. Note: Baseline established at initial presentation (August 2023). Follow-up timepoints correspond to months post-initiation of multimodal rehabilitation (September 2023). The 18-month and 24-month assessments were both conducted in September 2024 due to scheduling; the 27-month assessment was conducted in October 2024.

## Data Availability

The raw data supporting the conclusions of this article will be made available by the authors on request.

## Abbreviations

The following abbreviations are used in this manuscript:

PPCS	Persistent post-concussive syndrome
mTBI	mild traumatic brain injury
CARE	CAse REports
DMN	default mode network
tPBM	Transcranial photobiomodulation
tVNS	Transcutaneous vagus nerve stimulation
HRV	heart rate variability
MRI	Magnetic Resonance Imaging
VOMS	The Vestibular/Ocular Motor Screening
vHIT	video head impulse testing
DHI	Dizziness Handicap Inventory
qEEG	Quantitative EEG
ERP	Event-related potential
SAC	Standardized Assessment of Concussion
UPSIT	The University of Pennsylvania Smell Identification Test
HIPAA	Health Insurance Portability and Accountability Act
